# First-Trimester Thyroid-Stimulating Hormone Levels and Gestational Diabetes Mellitus: A Retrospective Cohort Study

**DOI:** 10.3390/metabo16100772

**Published:** 2026-10-13

**Authors:** Büşra Şahin, Tansu Bahar Gürbüz, Fazilet Tatar, Rümeysa Uzun, Melike Özyurt Topuzoğlu, Ramazan Erda Pay, Yaprak Üstün

**Affiliations:** 1 Department of Obstetrics and Gynecology, Kocaeli City Hospital, University of Health Sciences, Kocaeli 41060, Türkiye; 2 Department of Obstetrics and Gynecology, Cerrahpaşa Faculty of Medicine, Istanbul University-Cerrahpaşa, Istanbul 34098, Türkiye; drtnsugurbuz@gmail.com; 3 Department of Obstetrics and Gynecology, Ankara Bilkent City Hospital, Ankara 06800, Türkiye; fazilet1907.ft@gmail.com; 4 Department of Obstetrics and Gynecology, Samsun City Hospital, Samsun 55080, Türkiye; dr.rumeysauzun@gmail.com; 5 Department of Obstetrics and Gynecology, Yozgat Boğazlıyan State Hospital, Yozgat 66400, Türkiye; melikeozyurt94@gmail.com; 6 Department of Obstetrics and Gynecology, Ankara Etlik City Hospital, Ankara 06800, Türkiye; drramazanpay@gmail.com; 7 Department of Obstetrics and Gynecology, İzmir City Hospital, İzmir 35540, Türkiye; yaprak.ustun@sbu.edu.tr

**Keywords:** thyroid-stimulating hormone, subclinical hypothyroidism, gestational diabetes mellitus, pregnancy, first trimester

## Abstract

**Background:** The clinical relevance of a mildly elevated first-trimester thyroid-stimulating hormone (TSH) concentration is uncertain, recent meta-analyses disagree, and few studies have directly contrasted a TSH of 2.5–4.0 mIU/L with a TSH above 4.0 mIU/L. **Methods:** In this single-centre retrospective cohort study, 218 singleton pregnancies with a first-trimester TSH ≥ 2.5 and ≤10.0 mIU/L and a concurrent free thyroxine (fT4) measurement were classified as high-normal TSH (2.5–4.0 mIU/L; *n* = 168) or elevated TSH (>4.0–10.0 mIU/L; *n* = 50; subclinical hypothyroidism in 47 and biochemically overt hypothyroidism in 3). The primary outcome was gestational diabetes mellitus (GDM); secondary outcomes were a composite obstetric complication, individual obstetric and neonatal outcomes, and first- and second-trimester screening markers. Two logistic regression models adjusted for maternal age were fitted; because levothyroxine was started after the index TSH measurement, it was treated as a potential intermediate and examined only in post hoc sensitivity analyses. **Results:** GDM occurred in 7/50 (14.0%) women with TSH > 4.0 mIU/L versus 6/168 (3.6%) with TSH 2.5–4.0 mIU/L (Fisher’s exact test, *p* = 0.013; unadjusted odds ratio (OR) 4.40, 95% confidence interval (CI) 1.40–13.76; risk difference 10.4 percentage points, 95% CI 0.4–20.4). After adjustment for maternal age, TSH > 4.0 mIU/L remained associated with GDM (adjusted OR 5.24, 95% CI 1.60–17.16; *p* = 0.006), an estimate based on only 13 events; additional adjustment for levothyroxine use gave a similar estimate (adjusted OR 4.51, 95% CI 1.33–15.22). The composite obstetric complication did not differ (32.0% vs. 20.2%; χ^2^(1) = 3.02, *p* = 0.082; adjusted OR 1.95, 95% CI 0.96–3.98), nor did individual obstetric outcomes, neonatal outcomes or screening markers. **Conclusions:** First-trimester TSH > 4.0 mIU/L was associated with a higher frequency of GDM. Given the few events, the wide confidence interval, unmeasured body-mass index and thyroid-peroxidase-antibody status and the absence of a TSH < 2.5 mIU/L comparison group, the finding is hypothesis-generating and does not establish 4.0 mIU/L as a clinically discriminatory threshold.

## 1. Introduction

Maternal thyroid function undergoes a marked physiological re-regulation during pregnancy. In early pregnancy, rising levels of human chorionic gonadotrophin (hCG) exert a weak agonist effect via the TSH receptor, thereby increasing thyroid hormone production; consequently, maternal TSH concentrations fall during this period. At the same time, the production of thyroxine (T4) and triiodothyronine (T3), along with the daily iodine requirement, increases by approximately 50%. Consequently, the interpretation of thyroid function tests in pregnant women cannot be based on the criteria used for non-pregnant women [1,2].

The fetal thyroid gland is not functional until the beginning of the second trimester; during this period, the fetus is dependent on maternal thyroid hormones for growth and neurodevelopment. The association between overt maternal thyroid disorders and adverse pregnancy outcomes is well established; however, there is no consensus regarding the clinical implications of mild abnormalities such as subclinical hypothyroidism [3,4].

TSH concentrations are influenced by gestational age, thyroid autoimmunity, ethnicity and population iodine status; population- and trimester-specific reference ranges are therefore recommended [1,5]. Because such ranges are unavailable in most laboratories, pragmatic alternatives have been evaluated: an individual participant meta-analysis could not identify any modification of non-pregnancy reference intervals that adequately approximated trimester-specific intervals [6], and full standardisation of TSH and fT4 to gestational age rather than to trimester did not improve, and in some cases weakened, their associations with clinical outcomes [7]. Against this background, the historical first-trimester upper limit of 2.5 mIU/L, introduced in 2011 [8], was shown to overestimate the prevalence of subclinical hypothyroidism, and a fixed limit of 4.0 mIU/L is now recommended when assay- and trimester-specific intervals are not available [1,3,9].

The evidence on the consequences of mild gestational thyroid dysfunction remains inconsistent. For hypertensive disorders, an individual-participant-data meta-analysis of 19 cohorts (46,528 women) reported a U-shaped relationship between maternal TSH and pre-eclampsia [10], whereas a large retrospective cohort linked a mildly elevated first-trimester TSH (2.5–4.08 mIU/L) to miscarriage and to a composite of maternal complications [11], and a meta-analysis found no association of subclinical hypothyroidism with preterm birth, although overt hypothyroidism and hyperthyroidism were associated with a small increase in risk [12]. For GDM, conventional meta-analyses of aggregate data have generally reported an increased risk in subclinical hypothyroidism (pooled odds ratios approximately 1.3–1.5) [13,14,15], although the association was not significant for subclinical hypothyroidism diagnosed in the first trimester or without thyroid antibodies [15], and Kent et al. showed that the association depends on the diagnostic threshold used and on thyroid-antibody status [16]. In contrast, the most recent and methodologically strongest source of evidence, an individual participant data meta-analysis of more than 50,000 pregnancies, found no association of TSH, subclinical hypothyroidism or thyroid autoimmunity with GDM, while isolated hypothyroxinaemia was associated with a higher risk [17]. Divergent TSH cut-offs, unreported autoantibody status, heterogeneous GDM criteria and variable treatment practices are the principal sources of this discrepancy.

Two specific gaps follow from this literature. First, almost all available studies compare women with subclinical hypothyroidism against a euthyroid reference group that also contains women with a TSH below 2.5 mIU/L; the clinically relevant question of whether outcomes differ between the high-normal band (2.5–4.0 mIU/L), in which no treatment is currently advised, and a TSH above 4.0 mIU/L has rarely been addressed directly. Second, most studies evaluate obstetric and neonatal outcomes in isolation and do not examine, in the same cohort, whether first- and second-trimester biochemical screening markers are also affected, even though these markers are obtained in routine care at the same gestational ages.

Thyroid hormones regulate hepatic glucose production, peripheral glucose uptake and pancreatic β-cell function [18,19], and pregnancy is accompanied by a physiological decline in insulin sensitivity [20]; it is therefore biologically plausible that a mild elevation of TSH in early pregnancy is accompanied by a less favourable metabolic adaptation. We hypothesised that, compared with women whose first-trimester TSH lies between 2.5 and 4.0 mIU/L, women with a TSH above 4.0 mIU/L have a higher frequency of GDM. The primary outcome was GDM; secondary outcomes were a composite obstetric complication, individual obstetric and neonatal outcomes, and first- and second-trimester biochemical screening markers. The intended contribution of this study is not to re-estimate the overall effect of subclinical hypothyroidism, which large individual participant analyses are far better powered to do, but to provide direct, contemporaneous data from an iodine-sufficient Turkish population on the specific 2.5–4.0 versus >4.0 mIU/L contrast, together with prenatal screening markers that are rarely reported alongside clinical outcomes.

## 2. Materials and Methods

### 2.1. Study Design, Setting and Reporting

This single-centre retrospective cohort study was conducted at Etlik Zübeyde Hanım Women’s Health Training and Research Hospital, Ankara, Türkiye. Pregnancies managed and delivered at the institution between 1 January 2018 and 1 December 2018 were screened. The study is reported in accordance with the Strengthening the Reporting of Observational Studies in Epidemiology (STROBE) statement for cohort studies [21]; the completed STROBE checklist is provided as Supplementary File S1.

### 2.2. Participants and Eligibility Criteria

**Figure 1:**
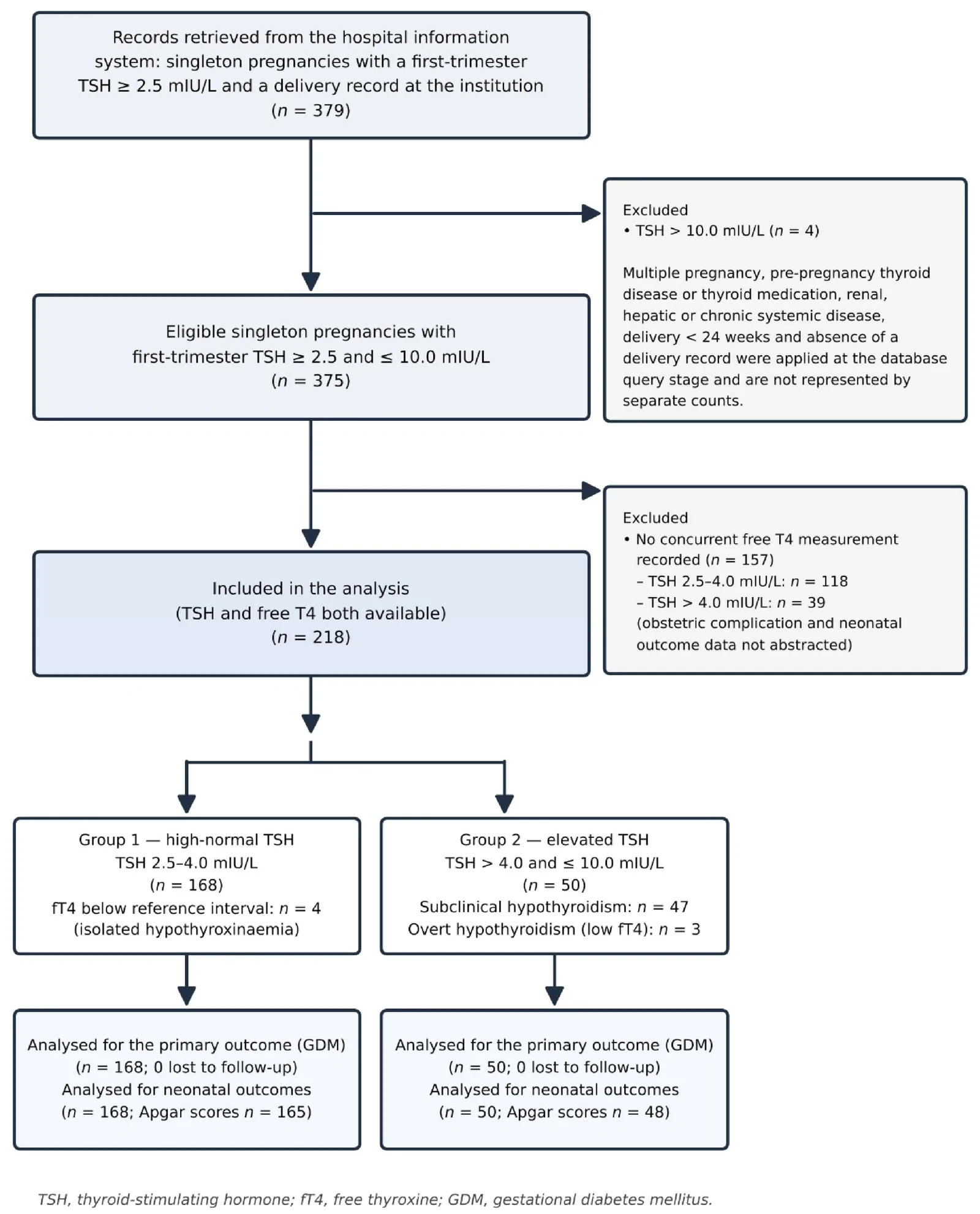
Flow of participants. TSH, thyroid-stimulating hormone; fT4, free thyroxine; GDM, gestational diabetes mellitus.

The hospital information system was queried for all consecutive singleton pregnancies in which a first-trimester thyroid function test had been performed, the serum TSH was ≥2.5 mIU/L and the delivery was recorded at the institution; this query returned 379 records. The delivery record available for every woman comprised the date and mode of delivery, birth weight and birth length. Pregnancies were required to have been delivered at ≥24 weeks’ gestation. Multiple pregnancy, a known thyroid disorder before pregnancy (Graves’ disease, autoimmune thyroiditis or other), use of thyroid replacement or antithyroid medication before pregnancy, renal failure, liver disease or another chronic systemic condition capable of affecting thyroid function, and the absence of a delivery record at the institution were exclusion criteria applied at the query stage, so that the records returned already satisfied them; separate counts for these criteria are consequently not available and are not shown in Figure 1.

Two further exclusions were then applied. Four women with a TSH > 10.0 mIU/L were excluded, because a TSH above this value is managed as overt hypothyroidism irrespective of fT4 [1], leaving 375 eligible women. Of these, 157 (41.9%) had no concurrent fT4 measurement recorded and were excluded, so that 218 women entered the analysis (Figure 1). Detailed obstetric-complication and neonatal variables (GDM and the other obstetric outcomes, Apgar scores, neonatal complications and levothyroxine treatment) were abstracted from the electronic medical record only for women with both TSH and fT4 available; for the 157 excluded women only the routinely structured delivery record was retrieved (Section 2.7). Women with a TSH < 2.5 mIU/L were not represented in the source query; the consequences of this restriction for interpretation are addressed in the Discussion.

### 2.3. Study Groups

Participants were divided into two groups according to the first-trimester serum TSH concentration: Group 1 (high-normal TSH), TSH 2.5–4.0 mIU/L (*n* = 168); Group 2 (elevated TSH), TSH > 4.0 and ≤10.0 mIU/L (observed range 4.05–9.94 mIU/L; *n* = 50). Seven women (4 in Group 1 and 3 in Group 2) had an fT4 below the lower limit of the laboratory reference interval (11.2 pmol/L). In the three such women in Group 2 (TSH 4.83–9.94 mIU/L), an elevated TSH combined with a low fT4 constitutes biochemically overt hypothyroidism [1,3]; Group 2 therefore comprises 47 women with subclinical hypothyroidism (elevated TSH with normal fT4) and three with overt hypothyroidism, and is referred to as the elevated-TSH group. In the four such women in Group 1 (TSH 2.52–3.79 mIU/L), TSH lay within the 4.0 mIU/L reference limit and the pattern corresponds to isolated hypothyroxinaemia [1,17]. All seven women were retained in the main analysis; post hoc sensitivity analyses excluded first the three women with overt hypothyroidism and then all seven women with a low fT4 (Section 2.9).

### 2.4. Laboratory Methods

Serum TSH, free thyroxine (fT4) and free triiodothyronine (fT3) were measured in the hospital’s central biochemistry laboratory on a Roche Cobas e801 analyser (Roche Diagnostics, Mannheim, Germany) using electrochemiluminescence immunoassays. The laboratory reference intervals were 0.27–4.20 mIU/L for TSH, 11.2–22.1 pmol/L for fT4 and 3.1–6.8 pmol/L for fT3. Intra- and inter-assay coefficients of variation were both <3%. Trimester-specific, population-based reference intervals were not available for this assay, and the manufacturer’s interval was therefore used, in line with current guidance [1,6]. Thyroid-peroxidase antibodies were not measured as part of routine care during the study period and are unavailable, as is the exact gestational week at which thyroid function was tested; both are addressed as limitations. fT3 was measured in only 142 of the 218 women (65.1%), and fT3 comparisons are therefore restricted to that subgroup. First- and second-trimester aneuploidy screening markers (free β-hCG, PAPP-A and AFP, with their multiples of the median (MoM)) were retrieved from the same laboratory information system were measured by chemiluminescent immunoassay on an IMMULITE 2000 analyser (Siemens Healthcare Diagnostics Products Ltd., Llanberis, UK).

### 2.5. Outcomes and Definitions

The primary outcome was GDM. All women were offered a 75 g oral glucose tolerance test between 24 weeks 0 days and 28 weeks 6 days as part of routine antenatal care, and GDM was diagnosed when at least one of the following thresholds was met: fasting plasma glucose ≥ 92 mg/dL, 1 h value ≥ 180 mg/dL, or 2 h value ≥ 153 mg/dL (IADPSG/WHO criteria) [22]. GDM status was extracted from the coded diagnosis field of the hospital record; the individual glucose values underlying each diagnosis were not retrievable from the database, and the completeness of screening could therefore not be verified for each participant. This is stated as a limitation.

Gestational hypertension was defined as a systolic blood pressure ≥ 140 mmHg and/or a diastolic blood pressure ≥ 90 mmHg arising after 20 weeks’ gestation in a woman with no prior history of hypertension, and pre-eclampsia as gestational hypertension accompanied by proteinuria (≥300 mg/24 h) or evidence of target-organ dysfunction [23]. Fetal growth restriction (FGR) was defined as a sonographic estimated fetal weight below the 10th percentile for gestational age [24]. Preterm birth was delivery before 37 weeks’ gestation, and preterm prelabour rupture of membranes (PPROM) was rupture of the membranes before the onset of labour and before 37 weeks [25]. Oligohydramnios and polyhydramnios were defined, respectively, as an amniotic fluid index < 5 cm and >24 cm (or a deepest vertical pocket < 2 cm and >8 cm) at any antenatal ultrasound examination.

The composite obstetric complication was defined as the occurrence of at least one of the following: GDM, gestational hypertension or pre-eclampsia, FGR, preterm birth or PPROM, oligohydramnios, or polyhydramnios. Each woman contributed only once to the composite, irrespective of the number of component events.

Neonatal outcomes were birth weight, birth length, and 1- and 5-min Apgar scores. The composite neonatal complication comprised at least one of the four conditions recorded in the database: respiratory distress, neonatal jaundice requiring treatment, prematurity-related morbidity, and major fetal anomaly or neonatal death. Congenital hypothyroidism, ascertained through the national newborn screening programme, was recorded separately and was documented for 209 of the 218 neonates (95.9%).

### 2.6. Covariates

Covariates were selected on clinical grounds and restricted in number because of the small number of outcome events. Maternal age, an established determinant of GDM [26] that precedes the exposure, was the only covariate in the primary models. Levothyroxine use during pregnancy was not included in the primary models. Treatment was initiated at the discretion of the treating clinician, generally after and in response to the index TSH measurement; it is therefore a post-exposure variable that may lie on the pathway between TSH and the outcomes rather than a baseline confounder, and adjustment for such a variable can remove part of the association of interest or introduce bias [27]. Additional adjustment for levothyroxine use, and restriction to untreated women, are reported as sensitivity analyses (Section 2.9). Levothyroxine status was not recorded for six women (three in each group), who were classified as untreated. Maternal body-mass index, parity, smoking, family history of diabetes and thyroid-peroxidase antibody status were not recorded in the source database and could not be included.

### 2.7. Data Sources and Missing Data

Demographic, obstetric, clinical and laboratory data were retrieved retrospectively from the hospital information management system and the electronic medical record. All analyses were complete-case analyses; no imputation was performed. The principal source of missing data was the absence of a concurrent fT4 measurement, which led to the exclusion of 157 of the 375 eligible women (41.9%); 118 of these had a TSH in the 2.5–4.0 mIU/L band and 39 a TSH above 4.0 mIU/L. Women excluded for a missing fT4 did not differ from those included in maternal age (median 26.7 vs. 27.4 years; Mann–Whitney U test, *p* = 0.155) or first-trimester TSH (median 3.18 vs. 3.17 mIU/L; *p* = 0.754). Because the delivery record was available for all 379 women, the excluded and included women could also be compared on delivery characteristics, and they did not differ in birth weight (median 3100 vs. 3148 g; U = 16627.5, z = −0.47, *p* = 0.640), birth length (median 50.0 vs. 50.0 cm; *p* = 0.784), caesarean delivery (51.6% vs. 52.8%; χ^2^(1) = 0.05, *p* = 0.824) or infant sex (male 45.9% vs. 51.8%; χ^2^(1) = 1.30, *p* = 0.254). The detailed obstetric-complication and neonatal variables, however, had not been abstracted for the excluded women: no obstetric complication was coded for any of them, Apgar scores and neonatal complications were blank, and these fields were therefore treated as not ascertained rather than as true absence of events. The excluded women could consequently not be compared with the analysed cohort with respect to GDM or other outcomes; this is acknowledged as a limitation. Within the analysed cohort, missing values were confined to fT3 (76/218), Apgar scores (5/218), levothyroxine status (6/218; classified as untreated, Section 2.6), congenital hypothyroidism status (9/218) and the biochemical screening markers; the number of available observations is reported alongside the corresponding results.

### 2.8. Sample Size

No a priori sample-size calculation was performed; all eligible records generated during the study period were analysed, and the sample size was therefore determined by data availability. A post hoc power calculation performed using IBM SPSS Statistics, version 27.0 (IBM Corp., Armonk, NY, USA), indicated that, with 168 and 50 women per group, a two-sided α of 0.05 and a GDM frequency of 3.6% in Group 1, the study had 80% power to detect a frequency of approximately 16% or higher in Group 2. The study was consequently underpowered for smaller differences and for rarer outcomes such as pre-eclampsia, preterm birth and FGR (for the observed difference in gestational hypertension/pre-eclampsia, post hoc power was 5%).

### 2.9. Statistical Analysis

All statistical analyses, including model diagnostics and post hoc sensitivity analyses, were performed using IBM SPSS Statistics, version 27.0 (IBM Corp., Armonk, NY, USA). The distribution of continuous variables was examined with the Kolmogorov–Smirnov and Shapiro–Wilk tests and with histograms and Q–Q plots. Normally distributed variables are presented as mean ± standard deviation (SD) and non-normally distributed variables as median (Q1–Q3); categorical variables are presented as *n* (%). Continuous variables were compared with the independent-samples t test or the Mann–Whitney U test as appropriate, and categorical variables with the Pearson χ^2^ test; Fisher’s exact test was used whenever any expected cell count was below five. Test statistics are reported with their standard symbols (t with degrees of freedom, U with the standardised z value, and χ^2^ with degrees of freedom), and all tests were two-sided with α = 0.05. Wilson confidence intervals are given for proportions.

Two multivariable binary logistic regression models were fitted, with GDM (Model A) and the composite obstetric complication (Model B) as dependent variables; in both, the TSH group (>4.0 mIU/L versus 2.5–4.0 mIU/L, the latter as reference) was the exposure of interest, and maternal age was entered as the only covariate, for the reasons given in Section 2.6. Model assumptions and stability were examined as follows: linearity of the logit for maternal age was tested with the Box–Tidwell procedure; multicollinearity with variance inflation factors; influential observations with Cook’s distance and leverage; calibration with the Hosmer–Lemeshow goodness-of-fit test; and discrimination with the area under the receiver-operating-characteristic curve (AUC). 

Model A was based on only 13 GDM events (6.5 events per estimated parameter), which is below the conventional minimum of 10 and predisposes to small-sample bias and inflated confidence intervals. The following sensitivity analyses were therefore performed for Model A; all were defined after the primary results were known, during peer review, and are reported as post hoc: (i) the unadjusted association between TSH group and GDM; (ii) additional adjustment for levothyroxine use; (iii) Firth’s penalised maximum-likelihood logistic regression, which reduces small-sample bias in sparse data; (iv) exclusion of the three women in Group 2 with biochemically overt hypothyroidism; (v) exclusion of all seven women with an fT4 below the reference interval (overt hypothyroidism in Group 2 and isolated hypothyroxinaemia in Group 1); and (vi) restriction to women not treated with levothyroxine, recognising that restriction on a post-exposure variable can itself introduce selection bias. Model B was additionally refitted with levothyroxine use as a covariate. In addition, the robustness of the primary adjusted association to unmeasured confounding was quantified using the E-value, defined as the minimum strength of association, on the risk-ratio scale, that an unmeasured confounder would need to have with both the exposure and the outcome to explain away the observed estimate. Because several secondary outcomes were examined, no formal correction for multiple comparisons was applied and all secondary analyses are regarded as exploratory.

### 2.10. Ethical Approval

The study protocol was approved by the Ethics Committee of Etlik Zübeyde Hanım Women’s Health Training and Research Hospital, Ankara, Türkiye (approval number: 11; date of approval: 6 September 2022). The same committee waived the requirement for written informed consent because of the retrospective design and the exclusive use of anonymised routine clinical data. All procedures were carried out in accordance with the World Medical Association Declaration of Helsinki (2013 revision) and with national legislation on the protection of personal data.

## 3. Results

### 3.1. Participants

The database query returned 379 singleton pregnancies with a first-trimester TSH ≥ 2.5 mIU/L. Four women were excluded because the TSH exceeded 10.0 mIU/L and 157 because no concurrent fT4 measurement was recorded, leaving 218 women for analysis: 168 (77.1%) in Group 1 (TSH 2.5–4.0 mIU/L) and 50 (22.9%) in Group 2 (TSH > 4.0–10.0 mIU/L). No woman was lost to follow-up, and delivery data were complete for all 218 participants (Figure 1).

### 3.2. Baseline Characteristics

Maternal age was comparable between the groups (median 27.3 vs. 27.9 years; U = 4539.0, z = 0.87, *p* = 0.387). By design, TSH was higher in Group 2 (median 4.57 vs. 2.96 mIU/L; U = 0.0, z = −10.73, *p* < 0.001), whereas fT4 (t(216) = −0.28, *p* = 0.781) and fT3 (t(140) = 0.87, *p* = 0.386) did not differ. Levothyroxine was prescribed during pregnancy more often in Group 2 (32.0% vs. 14.3%; χ^2^(1) = 8.07, *p* = 0.004). The caesarean delivery rate was similar (54.0% vs. 52.4%; χ^2^(1) = 0.04, *p* = 0.840). Full details are given in Table 1.

### 3.3. Primary Outcome: Gestational Diabetes Mellitus

GDM was diagnosed in 7 of 50 women in Group 2 (14.0%, 95% CI 7.0–26.2) and in 6 of 168 women in Group 1 (3.6%, 95% CI 1.6–7.6; Fisher’s exact test, *p* = 0.013). The unadjusted odds ratio for Group 2 versus Group 1 was 4.40 (95% CI 1.40–13.76) and the absolute risk difference was 10.4 percentage points (95% CI 0.4–20.4). Thirteen GDM events were observed in total (Table 2).

### 3.4. Other Obstetric Outcomes

The composite obstetric complication occurred in 16 of 50 women in Group 2 (32.0%) and 34 of 168 in Group 1 (20.2%), a difference that did not reach statistical significance (χ^2^(1) = 3.02, *p* = 0.082; OR 1.85, 95% CI 0.92–3.75). None of the individual components other than GDM differed between the groups, and all confidence intervals for these components were wide and included the null value (Table 2).

### 3.5. Neonatal Outcomes

Birth weight, birth length, and 1- and 5-min Apgar scores were similar in the two groups. The composite neonatal complication occurred in 12 of 168 neonates in Group 1 (7.1%) and 1 of 50 in Group 2 (2.0%; Fisher’s exact test, *p* = 0.307; OR 0.27, 95% CI 0.03–2.09); in Group 1 these comprised neonatal jaundice (*n* = 6), respiratory distress (*n* = 3), prematurity-related morbidity (*n* = 2) and one major fetal anomaly with neonatal death, and in Group 2 a single case of respiratory distress. No case of congenital hypothyroidism was recorded among the 209 neonates for whom screening status was documented (Table 3).

### 3.6. First- and Second-Trimester Biochemical Screening Markers

Free β-hCG, β-hCG MoM, PAPP-A, PAPP-A MoM, AFP and AFP MoM were similar in the two groups (all *p* ≥ 0.14). These markers were available only in the subgroup of women who underwent aneuploidy screening, and the denominators for each marker are given in Table 4.

### 3.7. Multivariable Analysis, Diagnostics and Sensitivity Analyses

**Figure 2:**
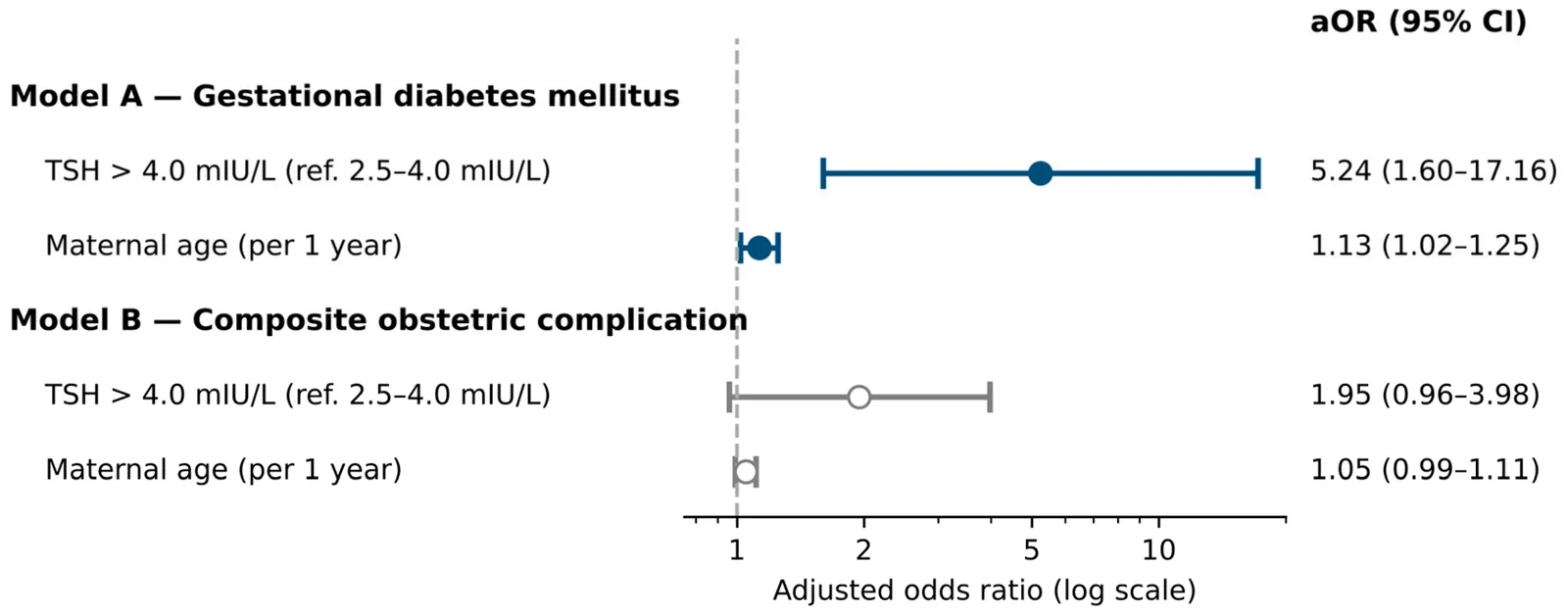
Adjusted odds ratios with 95% confidence intervals from the two multivariable logistic regression models. Filled circles denote variables for which the confidence interval excludes 1; open circles denote variables for which it does not. The dashed vertical line marks the null value (odds ratio = 1) and the x-axis is plotted on a logarithmic scale. Model A was based on 13 outcome events and Model B on 50; both were adjusted for maternal age only. aOR, adjusted odds ratio; CI, confidence interval; TSH, thyroid-stimulating hormone.

In Model A, TSH > 4.0 mIU/L remained associated with GDM after adjustment for maternal age (adjusted OR 5.24, 95% CI 1.60–17.16; Wald χ^2^(1) = 7.50, *p* = 0.006), as did maternal age (adjusted OR 1.13 per year, 95% CI 1.02–1.25; Wald χ^2^(1) = 5.67, *p* = 0.017). In Model B, the association between TSH group and the composite obstetric complication was not statistically significant (adjusted OR 1.95, 95% CI 0.96–3.98; Wald χ^2^(1) = 3.39, *p* = 0.066), and neither was maternal age (adjusted OR 1.05 per year, 95% CI 0.99–1.11; *p* = 0.079) (Table 5 and Table 6; Figure 2).

Model diagnostics were satisfactory. The Box–Tidwell test indicated no violation of the linearity of the logit for maternal age (*p* = 0.151 in Model A and *p* = 0.899 in Model B); the largest variance inflation factor was 1.00, indicating no relevant multicollinearity; the maximum Cook’s distance was 0.12 in Model A and 0.04 in Model B, with maximum leverage 0.12 and 0.06, so no single observation was unduly influential; the Hosmer–Lemeshow test indicated adequate calibration (χ^2^(8) = 5.67, *p* = 0.684 for Model A and χ^2^(8) = 9.05, *p* = 0.338 for Model B); and discrimination was moderate for Model A (AUC 0.772, 95% CI 0.619–0.925) and poor for Model B (AUC 0.603, 95% CI 0.510–0.695). Model A nevertheless rests on 13 events and 6.5 events per estimated parameter, and the width of the confidence interval for the exposure (a more than ten-fold span from 1.60 to 17.16) reflects this sparsity.

The post hoc sensitivity analyses were directionally consistent with the primary model (Table 5). The unadjusted odds ratio was 4.40 (95% CI 1.40–13.76). Additional adjustment for levothyroxine use gave an adjusted odds ratio of 4.51 (95% CI 1.33–15.22; *p* = 0.015), with levothyroxine use itself not significantly associated with GDM (adjusted OR 3.04, 95% CI 0.91–10.14; *p* = 0.070). Firth’s penalised logistic regression, which corrects small-sample bias, gave 5.00 (95% CI 1.60–15.64; *p* = 0.006). Excluding the three women with overt hypothyroidism gave 5.58 (95% CI 1.70–18.29; *p* = 0.005), and excluding all seven women with a low fT4 gave 5.41 (95% CI 1.65–17.74; *p* = 0.005). Restricted to the 178 women not treated with levothyroxine, among whom only 7 GDM events occurred, the point estimate was similar but imprecise (adjusted OR 3.82, 95% CI 0.77–18.85; *p* = 0.100). In Model B, additional adjustment for levothyroxine use gave an adjusted odds ratio for the TSH group of 1.63 (95% CI 0.78–3.42; *p* = 0.197), while levothyroxine use itself was associated with the composite outcome (adjusted OR 2.80, 95% CI 1.32–5.94; *p* = 0.007) (Table 6). The E-value for the primary adjusted estimate was 9.95, and that for the confidence limit closest to the null was 2.58: an unmeasured confounder (most plausibly maternal body-mass index or obesity) would have to be associated with both TSH > 4.0 mIU/L and GDM by a risk ratio of at least 2.58, above and beyond maternal age, for the lower confidence limit to be moved to the null. Obesity is an established risk factor for GDM [22] and has been associated with thyroid function test abnormalities in pregnancy, although this association was modest in an individual participant data meta-analysis [28]; residual confounding therefore remains a plausible explanation for part of the observed association.

## 4. Discussion

In this retrospective cohort of 218 singleton pregnancies, GDM was about four times more frequent among women whose first-trimester TSH exceeded 4.0 mIU/L than among women in the high-normal 2.5–4.0 mIU/L band, and the association persisted after adjustment for maternal age and was directionally consistent across post hoc sensitivity analyses, including additional adjustment for levothyroxine use and exclusion of women with a low fT4. Neither the composite obstetric complication nor any individual obstetric or neonatal outcome differed between the groups, and first- and second-trimester screening markers were similar. Because the adjusted estimate is based on 13 events and has a confidence interval spanning a more than ten-fold range, this result should be read as a signal that requires confirmation rather than as a quantitative estimate of effect.

The optimal upper limit for TSH in pregnancy remains debated [1,3,5,9]. The fixed limit of 2.5 mIU/L introduced in 2011 [8] substantially inflated the apparent prevalence of subclinical hypothyroidism; in a Turkish population the reported prevalence rose from 2.3% to 22.3% when this threshold was applied [29]. Contemporary guidance therefore recommends 4.0 mIU/L when assay- and trimester-specific intervals are unavailable [1], and individual-participant analyses have shown that neither modified non-pregnancy intervals nor trimester-based categorisation captures early-pregnancy thyroid physiology precisely [6,7]. Our data are compatible with a difference in GDM frequency on either side of the 4.0 mIU/L cut-off, but they cannot establish that this cut-off is discriminatory: we did not evaluate alternative thresholds, we had no women with a TSH < 2.5 mIU/L, and a single dichotomy examined in one small cohort cannot validate a threshold.

Our finding sits within a genuinely divided literature. Aggregate-data meta-analyses have mostly reported a moderately increased risk of GDM in subclinical hypothyroidism [13,14,15], and Kent et al. found that the association is contingent on the diagnostic criteria applied and on thyroid-antibody status [16]. The largest and methodologically strongest analysis to date, however, an individual participant data meta-analysis by Osinga et al., found no association between TSH, subclinical hypothyroidism or thyroid autoimmunity and GDM, while isolated hypothyroxinaemia and lower fT4 were associated with a higher risk [17]. An observational study of our size cannot adjudicate between these positions, and the difference in magnitude between our point estimate and the pooled estimates of individual-participant analyses is more likely to reflect sampling variability, residual confounding and the particular comparison group we used than a true difference in biology.

Thyroid hormones influence pancreatic β-cell function, hepatic glucose production, peripheral glucose utilisation and insulin sensitivity [18]. Hepatic thyroid hormone signalling improves systemic insulin sensitivity through pathways that are partly independent of the classical thyroid hormone receptor β [19], and pregnancy is characterised by a progressive, physiological fall in insulin sensitivity that is normally compensated by increased β-cell secretion; in women who develop GDM this compensation is insufficient [20]. Within this framework, a mild elevation of TSH could plausibly accompany a less favourable metabolic adaptation. Related clinical observations, however, largely concern fT4 and fT3 rather than TSH: maternal hypothyroxinaemia, but not subclinical hypothyroidism, has been associated with obesity and adverse metabolic parameters [30]; in a longitudinal study, higher fT3 and fT3/fT4 ratio, but neither TSH nor fT4, were associated with GDM risk [31]; and adverse outcomes were more frequent in women with subclinical hypothyroidism and concomitant first-trimester dyslipidaemia [32]. We emphasise, however, that no measure of insulin sensitivity, body composition or lipid status was available in our cohort, so these mechanisms are offered as context rather than as an explanation for our finding.

Maternal age was also associated with GDM in the adjusted model, each additional year corresponding to a 13% increase in the odds (OR 1.13, 95% CI 1.02–1.25). This is directionally consistent with, though larger than, the approximately 7.9% increase per year reported in a meta-analysis of more than 120 million participants [26]; the difference is readily explained by the imprecision of our estimate, whose confidence interval includes the pooled value.

No individual obstetric outcome other than GDM differed between the groups, and the composite obstetric complication did not differ either. The literature is also heterogeneous here: a large cohort of levothyroxine-treated women with subclinical hypothyroidism found no excess of gestational hypertension, preterm birth or other adverse maternal outcomes apart from GDM [33], and a meta-analysis found no association between subclinical hypothyroidism and preterm birth [12], whereas a large retrospective cohort linked a mildly elevated first-trimester TSH to miscarriage and maternal complications [11] and the individual-participant analysis by Toloza et al. identified a U-shaped relationship between maternal TSH and pre-eclampsia [10]. Our study was not designed to address these outcomes: with 9 cases of gestational hypertension or pre-eclampsia and 5 preterm births, post hoc power for the observed differences was approximately 5%, and the absence of a statistically significant difference should not be read as evidence of absence. The composite outcome, which aggregates conditions with different pathophysiology and very different frequencies and of which GDM is itself a component, adds little beyond the primary analysis and we interpret it cautiously.

In the sensitivity model additionally adjusted for levothyroxine, treatment was associated with the composite obstetric complication (adjusted OR 2.80, 95% CI 1.32–5.94). This should not be read as a harmful effect of treatment. Treatment was not randomised: it was initiated on clinical grounds after the index TSH measurement and was more than twice as common in the elevated-TSH group (32.0% vs. 14.3%), so the association most plausibly reflects confounding by indication, with levothyroxine acting as a marker of a higher baseline TSH and of greater clinician concern. Because treatment began after, and in response to, the exposure measurement, levothyroxine use is better regarded as a post-exposure and potentially intermediate variable than as a baseline confounder, and adjusting for it could bias the estimate for the TSH group [27]; this is why it was excluded from the primary models. Its inclusion changed the GDM estimate only modestly (adjusted OR 4.51 vs. 5.24), and restriction to untreated women gave a similar but imprecise point estimate (adjusted OR 3.82, 95% CI 0.77–18.85), based on 7 events. Randomised evidence does not indicate that levothyroxine increases obstetric complications: meta-analyses and an umbrella review of randomised trials report no effect of levothyroxine on GDM, with benefit for pregnancy loss and preterm delivery in some analyses [34,35]. Observational findings are less consistent: a propensity-score-matched analysis of women with a TSH between 2.5 and 10 mIU/L found a lower risk of pregnancy loss but a higher risk of small-for-gestational-age infants with treatment [36], and in a large retrospective cohort, treatment of women with a TSH of 2.5–4.08 mIU/L was associated with a higher risk of GDM [11].

Neonatal outcomes did not differ between the groups. The individual-participant analysis by Derakhshan et al. found only modest associations between maternal thyroid function and birthweight [37], and Maraka et al. reported non-significant increases in low birth weight, low Apgar score and small-for-gestational-age infants in subclinical hypothyroidism [4]; a retrospective cohort of treated subclinical hypothyroidism similarly reported broadly reassuring neonatal outcomes [33]. With 13 composite neonatal events in our cohort, our comparisons were underpowered.

First-trimester free β-hCG, β-hCG MoM, PAPP-A and PAPP-A MoM, and second-trimester AFP and AFP MoM did not differ between the groups, consistent with a previous report of no correlation between maternal thyroid function and first-trimester free β-hCG or PAPP-A [38], although differences in PAPP-A and β-hCG MoM values have been described in women with diagnosed hypothyroidism [39]. This is a practically useful negative finding: it suggests that a mildly raised first-trimester TSH does not require reinterpretation of aneuploidy screening results, although the subgroups with available markers comprised only about 80% of the cohort.

### 4.1. Strengths and Limitations

The principal strength of this study is the direct comparison of the 2.5–4.0 mIU/L and >4.0 mIU/L bands, which corresponds to the clinical decision currently faced when assay-specific reference intervals are unavailable and which most previous studies have not isolated. Thyroid function was assessed in early pregnancy; follow-up to delivery was complete for all 218 women; maternal, obstetric, neonatal and prenatal biochemical screening outcomes were analysed within a single cohort; and the primary association was directionally consistent across post hoc sensitivity analyses, including penalised regression, additional adjustment for levothyroxine use and exclusion of women with a low fT4.

The limitations are substantial and constrain interpretation. First, the design is single-centre and retrospective, so no causal inference is possible and generalisability to populations with different ethnic, geographical, socio-economic and iodine-status characteristics is uncertain. Second, the primary analysis rests on only 13 GDM events; with 6.5 events per estimated parameter the multivariable model is at risk of small-sample bias, and the confidence interval (1.60–17.16) is wide enough that the data are compatible with anything from a weak to a very strong association. Third, maternal body-mass index and thyroid-peroxidase-antibody status were not recorded; obesity is an established risk factor for GDM [22], has been associated with thyroid function test abnormalities [28] and is therefore a strong candidate for residual confounding; and the computed E-value of 2.58 for the confidence limit shows that a confounder of moderate strength would suffice to render the association non-significant. Parity, smoking and family history of diabetes were likewise unavailable. Fourth, levothyroxine was prescribed non-randomly and after the exposure measurement; we therefore treated it as a potential intermediate rather than a confounder, but its role cannot be fully resolved with these data, and the analysis restricted to untreated women was underpowered. Fifth, 157 of 375 eligible women (41.9%) were excluded because no concurrent fT4 measurement was recorded; although they did not differ from the analysed cohort in maternal age, TSH, birth weight, birth length or caesarean rate, their obstetric-complication and neonatal variables had not been abstracted, so outcome-related selection bias cannot be excluded. Sixth, the source query contained no women with a TSH < 2.5 mIU/L, so the 2.5–4.0 mIU/L group is a high-normal band rather than a true euthyroid reference, and our results say nothing about how either group compares with women below 2.5 mIU/L, a comparison that several guidelines regard as the relevant one. Seventh, thyroid function was measured once and the exact gestational week of sampling was not recorded, so neither the persistence of the TSH elevation nor the gestational-age dependence of the reference interval could be taken into account [7]. Eighth, GDM status was taken from coded diagnoses rather than from protocol-driven testing with retrievable glucose values, so misclassification is possible. Finally, multiple secondary outcomes were examined without correction for multiplicity, and the study was underpowered for all of them.

### 4.2. Clinical Implications

These findings do not justify a change to current screening or treatment practice. They are compatible with, but do not demonstrate, a higher metabolic risk among women whose first-trimester TSH exceeds 4.0 mIU/L, and they cannot be used to argue that this value is a clinically validated threshold. Universal GDM screening at 24–28 weeks is already recommended for all pregnant women [22], so no additional testing follows from our results. At most, a mildly raised first-trimester TSH may be regarded as one of several considerations prompting attention to modifiable metabolic risk factors, pending confirmation in adequately powered studies that adjust for body-mass index and thyroid autoimmunity.

### 4.3. Future Directions

Prospective multicentre studies with pre-specified sample sizes are required, incorporating maternal body-mass index, thyroid-peroxidase-antibody status, iodine status and metabolic markers, and using assay- and gestational-age-specific reference intervals rather than fixed cut-offs [7]. Serial thyroid function testing would allow persistent elevation to be distinguished from transient elevation, and the evaluation of several TSH strata, including values below 2.5 mIU/L, within one population would permit the shape of the relationship with metabolic risk to be characterised rather than assumed.

## 5. Conclusions

In this single-centre retrospective cohort, a first-trimester TSH above 4.0 mIU/L was associated with a higher frequency of gestational diabetes mellitus than a TSH of 2.5–4.0 mIU/L, and the association persisted after adjustment for maternal age and was directionally consistent across post hoc sensitivity analyses, including additional adjustment for levothyroxine use. Other obstetric and neonatal outcomes and prenatal biochemical screening markers were similar between the groups. Given that the estimate derives from 13 outcome events, that its confidence interval is wide, that maternal body-mass index and thyroid autoimmunity were unmeasured and that no comparison group with a TSH below 2.5 mIU/L was available, this association should be regarded as hypothesis-generating. It does not establish an independent or causal role for mildly elevated TSH, nor does it validate 4.0 mIU/L as a clinically discriminatory threshold; confirmation in larger prospective studies with full confounder adjustment is required before any clinical inference is drawn.

## Figures and Tables

**Table 1:** Demographic and laboratory characteristics of the study groups.

Variable	Group 1 TSH 2.5–4.0 mIU/L (*n* = 168)	Group 2 TSH > 4.0–10.0 mIU/L (*n* = 50)	Effect Estimate (95% CI)	Test Statistic	*p*
Maternal age, years	27.31 (24.40–33.28)	27.89 (22.60–31.82)	–	U = 4539.0, z = 0.87	0.387
TSH, mIU/L	2.96 (2.73–3.36)	4.57 (4.26–5.60)	–	U = 0.0, z = −10.73	<0.001
fT4, pmol/L	14.18 ± 1.59	14.26 ± 2.00	−0.08 (−0.61 to 0.46) a	t(216) = −0.28	0.781
fT3, pmol/L b	4.96 ± 0.60	4.86 ± 0.77	0.11 (−0.14 to 0.35) a	t(140) = 0.87	0.386
Levothyroxine use in pregnancy, *n* (%)	24 (14.3)	16 (32.0)	2.82 (1.35 to 5.89) c	Χ^2^(1) = 8.07	0.004
Caesarean delivery, *n* (%)	88 (52.4)	27 (54.0)	1.07 (0.57 to 2.01) c	Χ^2^(1) = 0.04	0.840

Data are median (Q1–Q3), mean ± standard deviation, or *n* (%). a Mean difference (Group 1 minus Group 2) with its 95% confidence interval. b fT3 was available for 106 women in Group 1 and 36 in Group 2 (142/218, 65.1%). Levothyroxine status was unrecorded for three women in each group, who were classified as untreated; all other variables were complete. c Odds ratio for Group 2 versus Group 1 with its 95% confidence interval. U, Mann–Whitney U statistic with the corresponding tie-corrected standardised z value; t, independent-samples t test with degrees of freedom in parentheses; χ^2^, Pearson chi-square test with degrees of freedom in parentheses; CI, confidence interval; fT3, free triiodothyronine; fT4, free thyroxine; TSH, thyroid-stimulating hormone.

**Table 2:** Maternal and obstetric outcomes by first-trimester TSH group.

Outcome	Group 1 (*n* = 168) *n* (%) [95% CI]	Group 2 (*n* = 50) *n* (%) [95% CI]	OR (95% CI) a	Test Statistic	*p*
Gestational diabetes mellitus	6 (3.6) [1.6–7.6]	7 (14.0) [7.0–26.2]	4.40 (1.40–13.76)	Fisher’s exact test	0.013
Gestational hypertension/pre-eclampsia	7 (4.2) [2.0–8.3]	2 (4.0) [1.1–13.5]	0.96 (0.19–4.77)	Fisher’s exact test	1.000
Fetal growth restriction	13 (7.7) [4.6–12.8]	6 (12.0) [5.6–23.8]	1.63 (0.58–4.53)	Fisher’s exact test	0.392
Preterm birth/PPROM	3 (1.8) [0.6–5.1]	2 (4.0) [1.1–13.5]	2.29 (0.37–14.11)	Fisher’s exact test	0.323
Oligohydramnios	6 (3.6) [1.6–7.6]	1 (2.0) [0.4–10.5]	0.55 (0.06–4.69)	Fisher’s exact test	1.000
Polyhydramnios	8 (4.8) [2.4–9.1]	2 (4.0) [1.1–13.5]	0.83 (0.17–4.06)	Fisher’s exact test	1.000
Composite obstetric complication b	34 (20.2) [14.9–26.9]	16 (32.0) [20.8–45.8]	1.85 (0.92–3.75)	Χ^2^(1) = 3.02	0.082

Data are *n* (%) with 95% Wilson confidence intervals for the proportion. Denominators are 168 and 50 for every outcome, with no missing values. a Odds ratio for Group 2 versus Group 1. b At least one of gestational diabetes mellitus, gestational hypertension or pre-eclampsia, fetal growth restriction, preterm birth or preterm prelabour rupture of membranes, oligohydramnios, or polyhydramnios; each woman contributes only once. Fisher’s exact test was used whenever an expected cell count was below five; χ^2^ denotes the Pearson chi-square test with degrees of freedom in parentheses. CI, confidence interval; OR, odds ratio; PPROM, preterm prelabour rupture of membranes; TSH, thyroid-stimulating hormone.

**Table 3:** Neonatal outcomes by first-trimester TSH group.

Outcome	*n* Analysed (Group 1/Group 2)	Group 1	Group 2	Effect Estimate (95% CI)	Test Statistic	*p*
Birth weight, g	168/50	3100 (2815–3465)	3190 (2918–3659)	–	U = 3653.0, z = −1.40	0.163
Birth length, cm	168/50	50.0 (48.0–51.0)	50.0 (48.3–52.0)	–	U = 3690.0, z = −1.32	0.189
Apgar score at 1 min	165/48	9.0 (9.0–9.0)	9.0 (9.0–9.0)	–	U = 3872.0, z = −0.71	0.480
Apgar score at 5 min	165/48	10.0 (10.0–10.0)	10.0 (10.0–10.0)	–	U = 3874.0, z = −0.69	0.490
Composite neonatal complication, *n* (%) a	168/50	12 (7.1) [4.1–12.1]	1 (2.0) [0.4–10.5]	0.27 (0.03–2.09) b	Fisher’s exact test	0.307
Congenital hypothyroidism, *n* (%)	160/49	0 (0)	0 (0)	Not estimable	Not applicable	–

Data are median (Q1–Q3) or *n* (%) with 95% Wilson confidence intervals. a Defined in Section 2.5. b Odds ratio for Group 2 versus Group 1. U, Mann–Whitney U statistic with the corresponding tie-corrected standardised z value; Fisher’s exact test was used because an expected cell count was below five. CI, confidence interval.

**Table 4:** First- and second-trimester biochemical screening markers by TSH group.

Marker	*n* Analysed (Group 1/Group 2)	Group 1	Group 2	Test Statistic	*p*
Free β-hCG, IU/L	137/37	34.30 (23.01–54.40)	38.30 (21.30–48.50)	U = 2571.0, z = 0.13	0.895
β-hCG, MoM	137/37	1.00 (0.69–1.58)	1.12 (0.67–1.52)	U = 2459.0, z = −0.28	0.783
PAPP-A, IU/L	138/37	2.47 (1.52–3.84)	2.30 (1.25–3.58)	U = 2774.0, z = 0.81	0.419
PAPP-A, MoM	138/37	0.95 (0.65–1.40)	0.74 (0.58–1.28)	U = 2957.0, z = 1.48	0.140
AFP, IU/mL	130/35	30.25 (24.15–39.90)	30.80 (24.45–41.15)	U = 2196.0, z = −0.31	0.756
AFP, MoM	130/34	0.99 (0.77–1.25)	1.07 (0.79–1.21)	U = 2125.0, z = −0.34	0.732

Data are median (Q1–Q3). All comparisons were made with the Mann–Whitney U test; U denotes the Mann–Whitney U statistic and z the corresponding tie-corrected standardised value. AFP, alpha-fetoprotein; β-hCG, beta human chorionic gonadotropin; MoM, multiple of the median; PAPP-A, pregnancy-associated plasma protein-A.

**Table 5:** Multivariable logistic regression for gestational diabetes mellitus (Model A) and sensitivity analyses.

Variable/Analysis	B (SE)	Adjusted OR (95% CI)	Wald χ^2^(1)	*p*
Primary model, adjusted for maternal age (*n* = 218; 13 events)				
TSH > 4.0 mIU/L (reference: 2.5–4.0 mIU/L)	1.657 (0.605)	5.24 (1.60–17.16)	7.50	0.006
Maternal age, per 1 year	0.121 (0.051)	1.13 (1.02–1.25)	5.67	0.017
Sensitivity analyses (TSH group)				
(i) Unadjusted	–	4.40 (1.40–13.76)	–	0.013 a
(ii) Additionally adjusted for levothyroxine use b	1.505 (0.621)	4.51 (1.33–15.22)	5.88	0.015
(iii) Firth penalised regression	1.610 (0.581)	5.00 (1.60–15.64)	7.67	0.006
(iv) Excluding overt hypothyroidism c	1.719 (0.606)	5.58 (1.70–18.29)	8.05	0.005
(v) Excluding all women with low fT4 d	1.688 (0.606)	5.41 (1.65–17.74)	7.77	0.005
(vi) Untreated women only e	1.339 (0.815)	3.82 (0.77–18.85)	2.70	0.100

Dependent variable: gestational diabetes mellitus. The primary model was adjusted for maternal age; all sensitivity analyses are post hoc. a Fisher’s exact test. b Adjusted for maternal age and levothyroxine use; levothyroxine status was unrecorded for six women, who were classified as untreated. c Excluding the three women in Group 2 with an fT4 below the laboratory reference interval, i.e., biochemically overt hypothyroidism (*n* = 215). d Excluding all seven women with an fT4 below the reference interval (three with overt hypothyroidism in Group 2 and four with isolated hypothyroxinaemia in Group 1; *n* = 211). e Restricted to the 178 women not treated with levothyroxine (7 events); adjusted for maternal age. Sensitivity analyses (iii)–(vi) were adjusted for maternal age. B, regression coefficient; CI, confidence interval; OR, odds ratio; SE, standard error; TSH, thyroid-stimulating hormone. Wald statistics are reported with one degree of freedom.

**Table 6:** Multivariable logistic regression for the composite obstetric complication (Model B).

Variable	B (SE)	Adjusted OR (95% CI)	Wald χ^2^(1)	*p*
TSH > 4.0 mIU/L (reference: 2.5–4.0 mIU/L)	0.669 (0.363)	1.95 (0.96–3.98)	3.39	0.066
Maternal age, per 1 year	0.049 (0.028)	1.05 (0.99–1.11)	3.08	0.079
Sensitivity analysis a: TSH > 4.0 mIU/L, additionally adjusted for levothyroxine use	0.488 (0.378)	1.63 (0.78–3.42)	1.67	0.197
Sensitivity analysis a: levothyroxine use in pregnancy	1.029 (0.384)	2.80 (1.32–5.94)	7.18	0.007

Dependent variable: composite obstetric complication (50 events among 218 women). The primary model was adjusted for maternal age. a Post hoc sensitivity model adjusted for maternal age and levothyroxine use (levothyroxine status unrecorded for six women, classified as untreated). Abbreviations as in Table 5.

## Data Availability

The raw data supporting the conclusions of this article will be made available by the corresponding author on reasonable request.

## Supplementary Materials

The following supporting information can be downloaded at: https://www.mdpi.com/article/10.3390/metabo16100772/s1. File S1: STROBE Statement—checklist of items that should be included in reports of cohort studies.

## Abbreviations

The following abbreviations are used in this manuscript:

AFP	alpha-fetoprotein
aOR	adjusted odds ratio
AUC	area under the receiver-operating-characteristic curve
β-hCG	beta human chorionic gonadotropin
CI	confidence interval
FGR	fetal growth restriction
fT3	free triiodothyronine
fT4	free thyroxine
GDM	gestational diabetes mellitus
IADPSG	International Association of the Diabetes and Pregnancy Study Groups
MoM	multiple of the median
OR	odds ratio
PAPP-A	pregnancy-associated plasma protein-A
PPROM	preterm prelabour rupture of membranes
SD	standard deviation
STROBE	Strengthening the Reporting of Observational Studies in Epidemiology
TSH	thyroid-stimulating hormone
WHO	World Health Organization
