# Supplementary material for: First-Trimester Thyroid-Stimulating Hormone Levels and Gestational Diabetes Mellitus: A Retrospective Cohort Study
Source: Metabolites. 2026 Oct 13;16(10):772. doi: 10.3390/metabo16100772 (PMC13706802; doi:10.3390/metabo16100772)
Supplement: Supplementary file 1 [file metabolites-16-00772-s001.zip › metabolites-4601231-supplementary.pdf]

Supplementary File S1. STROBE Statement — checklist of items that should be included in reports of cohort studies

Manuscript metabolites-4601231 — "First-Trimester Thyroid-Stimulating Hormone Levels and Gestational Diabetes Mellitus: A Retrospective Cohort Study". Locations refer to the revised manuscript.

Section/Topic	Item	Recommendation	Reported in (revised manuscript)
**Title and abstract**	1a	Indicate the study's design with a commonly used term in the title or the abstract	Title; Abstract, Methods sentence 1 ("single-centre retrospective cohort study")
	1b	Provide in the abstract an informative and balanced summary of what was done and what was found	Abstract (structured: Background, Methods, Results, Conclusions), including the number of events and the statement that the finding is hypothesis-generating
**Introduction**	2	Explain the scientific background and rationale for the investigation being reported	Section 1, paragraphs 1–5
	3	State specific objectives, including any prespecified hypotheses	Section 1, paragraph 6 (hypothesis; primary and secondary outcomes)
**Methods**	4	Present key elements of study design early in the paper	Section 2.1
	5	Describe the setting, locations, and relevant dates, including periods of recruitment, exposure, follow-up, and data collection	Section 2.1
	6a	Give the eligibility criteria, and the sources and methods of selection of participants. Describe methods of follow-up	Sections 2.2 and 2.7; Figure 1
	6b	For matched studies, give matching criteria and number of exposed and unexposed	Not applicable; the cohort was not matched
	7	Clearly define all outcomes, exposures, predictors, potential confounders, and effect modifiers. Give diagnostic criteria, if applicable	Sections 2.3 (exposure), 2.5 (all outcomes, including the composite obstetric and composite neonatal outcomes), 2.6 (covariates)
	8	For each variable of interest, give sources of data and details of methods of assessment (measurement). Describe comparability of assessment methods if there is more than one group	Sections 2.4 (laboratory methods, single assay platform for both groups) and 2.7 (data sources)
	9	Describe any efforts to address potential sources of bias	Sections 2.6 (levothyroxine treated as a post-exposure, potentially intermediate variable and excluded from the primary models), 2.7 (comparison of included and excluded women), 2.9 (sensitivity analyses and E-value); Section 4.1
	10	Explain how the study size was arrived at	Section 2.8 (no a priori calculation; all eligible records analysed; post hoc minimum detectable difference of about 16% in Group 2 at 80% power)
	11	Explain how quantitative variables were handled in the analyses. If applicable, describe which groupings were chosen and why	Sections 2.3 (TSH categorised at 4.0 mIU/L, with the rationale and the thyroid-function classification of women with low fT4) and 2.9 (maternal age as the only covariate); Tables 5 and 6 (maternal age modelled continuously, per 1-year increment); Section 3.7 (linearity of the logit for maternal age assessed with the Box–Tidwell test)

	12a	Describe all statistical methods, including those used to control for confounding	Section 2.9; model diagnostics (linearity of the logit, multicollinearity, influential observations, calibration and discrimination) reported in Section 3.7
	12b	Describe any methods used to examine subgroups and interactions	Not applicable; no subgroup or interaction analyses were performed
	12c	Explain how missing data were addressed	Section 2.7 (complete-case analysis; no imputation; included-versus-excluded comparison of maternal age, TSH and delivery characteristics); Section 2.6 (levothyroxine status unrecorded for six women, classified as untreated)
	12d	If applicable, explain how loss to follow-up was addressed	Section 3.1 and Figure 1: no participant was lost to follow-up (n = 218)
	12e	Describe any sensitivity analyses	Section 2.9 and Table 5 (all post hoc: unadjusted estimate; additional adjustment for levothyroxine; Firth penalised regression; exclusion of overt hypothyroidism; exclusion of all women with low fT4; restriction to untreated women; E-value) and Table 6
**Results**	13a	Report numbers of individuals at each stage of the study	Section 3.1 and Figure 1
	13b	Give reasons for non-participation at each stage	Figure 1; Sections 2.2 and 2.7
	13c	Consider use of a flow diagram	Figure 1
	14a	Give characteristics of study participants and information on exposures and potential confounders	Section 3.2; Table 1
	14b	Indicate number of participants with missing data for each variable of interest	Section 2.7; Tables 3 and 4 ("n analysed" columns); Table 1 footnote (free T3 142/218; levothyroxine status unrecorded for 6/218); Figure 1
	14c	Summarise follow-up time	Follow-up extended from first-trimester thyroid testing to delivery (Sections 2.2 and 3.1); it was complete for all 218 women (Section 3.1; Figure 1)
	15	Report numbers of outcome events or summary measures over time	Sections 3.3–3.6; Tables 2–4; Table 5 (13 events, primary-model row) and Table 6 footnote (50 events)
	16a	Give unadjusted estimates and, if applicable, confounder-adjusted estimates and their precision. Make clear which confounders were adjusted for and why they were included	Tables 2 and 3 (unadjusted odds ratios with 95% CI); Tables 5 and 6 and Figure 2 (adjusted estimates); Section 2.6 (rationale for each covariate)
	16b	Report category boundaries when continuous variables were categorized	Sections 2.2 and 2.3 (TSH ≥2.5 and ≤10.0 mIU/L; cut-point at 4.0 mIU/L)
	16c	If relevant, consider translating estimates of relative risk into absolute risk for a meaningful time period	Section 3.3 (absolute risk difference 10.4 percentage points, 95% CI 0.4–20.4)
	17	Report other analyses done — e.g. analyses of subgroups and interactions, and sensitivity analyses	Section 3.7 and Tables 5 and 6 (model diagnostics; six post hoc sensitivity analyses for Model A and additional adjustment for levothyroxine in Model B; E-value)
**Discussion**	18	Summarise key results with reference to study objectives	Section 4, paragraph 1
	19	Discuss limitations of the study, taking into account sources of potential bias or imprecision. Discuss both direction and magnitude of any potential bias	Section 4.1 (nine limitations, with the E-value used to quantify the strength of unmeasured confounding required to explain the association away)

	20	Give a cautious overall interpretation of results considering objectives, limitations, multiplicity of analyses, results from similar studies, and other relevant evidence	Sections 4 and 5 (finding presented as hypothesis-generating; multiplicity acknowledged in Section 2.9)
	21	Discuss the generalisability (external validity) of the study results	Section 4.1 (single-centre design; uncertain generalisability to populations with different ethnic, geographical, socio-economic and iodine-status characteristics; restricted TSH range) and Section 1, paragraph 6 (iodine-sufficient Turkish population)
**Other information**	22	Give the source of funding and the role of the funders for the present study and, if applicable, for the original study on which the present article is based	Funding statement

Note: An Explanation and Elaboration article discusses each checklist item and gives methodological background and published examples of transparent reporting. The STROBE checklist is best used in conjunction with that article, which is freely available at https://www.strobe-statement.org.
